# Advances in artificial intelligence, robotics, augmented and virtual reality in neurosurgery

**DOI:** 10.3389/fsurg.2023.1241923

**Published:** 2023-08-24

**Authors:** Kimia Kazemzadeh, Meisam Akhlaghdoust, Alireza Zali

**Affiliations:** ^1^ Students’ Scientific Research Center, Tehran University of Medical Sciences, Tehran, Iran; ^2^ Network of Neurosurgery and Artificial Intelligence (NONAI), Universal Scientific Education and Research Network (USERN), Tehran, Iran; ^3^ Functional Neurosurgery Research Center, Shohada Tajrish Comprehensive Neurosurgical Center of Excellence, Shahid Beheshti University of Medical Sciences, Tehran, Iran; ^4^ USERN Office, Functional Neurosurgery Research Center, Shahid Beheshti University of Medical Sciences, Tehran, Iran

**Keywords:** neurosurgery, artificial intelligence, augmented reality, robotics, virtual reality

## Abstract

Neurosurgical practitioners undergo extensive and prolonged training to acquire diverse technical proficiencies, while neurosurgical procedures necessitate a substantial amount of pre-, post-, and intraoperative clinical data acquisition, making decisions, attention, and convalescence. The past decade witnessed an appreciable escalation in the significance of artificial intelligence (AI) in neurosurgery. AI holds significant potential in neurosurgery as it supplements the abilities of neurosurgeons to offer optimal interventional and non-interventional care to patients by improving prognostic and diagnostic outcomes in clinical therapy and assisting neurosurgeons in making decisions while surgical interventions to enhance patient outcomes. Other technologies including augmented reality, robotics, and virtual reality can assist and promote neurosurgical methods as well. Moreover, they play a significant role in generating, processing, as well as storing experimental and clinical data. Also, the usage of these technologies in neurosurgery is able to curtail the number of costs linked with surgical care and extend high-quality health care to a wider populace. This narrative review aims to integrate the results of articles that elucidate the role of the aforementioned technologies in neurosurgery.

## Introduction

In contemporary society, artificial intelligence (AI) is widely perceived as an integral facet of human existence and has assumed a substantial function in the realm of medicine, encompassing domains such as diagnosis, prognosis, and treatment in recent decades. AI, in essence, represents the emulation of human cognitive faculties by machines, particularly computer systems, and was initially conceptualized in 1950. The emergence of deep learning (DL) and machine learning (ML) has provided a newfound opportunity to leverage personalized medicine and has concomitantly augmented the utilization of AI in medical procedures (1, 2).

Other technologies can be used in medicine as well. For instance, the field of Robotics is characterized by rapid progression, which is concurrently accompanied by advancements in AI and ML, ultimately leading to a metamorphosis of the medical practice (3). Augmented reality (AR) technology serves to enhance the physical world by rendering visible data that would otherwise be imperceptible to the human eye. In comparison to its virtual reality (VR) counterpart, AR technology boasts superior flexibility, albeit with a caveat of incomplete immersion on the part of the patient and physician. VR technology, on the other hand, entails complete submersion into a virtual environment facilitated by specialized equipment. In this scenario, the patient or physician is afforded the most comprehensive visualization attainable, only restricted by the boundaries of the virtual world (4).

Neurosurgery is an arduous vocation that demands a plethora of skills and attributes from its practitioners. To achieve success in this field, neurosurgeons must undergo extensive training, exhibit an appropriate degree of manual dexterity, possess acceptable hand-eye coordination, effectively engage in decision-making processes, show compassion, communicate well with patients and their families, and work well within a team (5). The efficacy and outcomes of surgical procedures are partially contingent upon the proficiency of the operating surgeon, leading to variations in patient experiences and results across different settings. While successful surgeries have the potential to produce advantageous outcomes for patients, errors can yield unfavorable consequences and, at times, even harmful effects (6). For example, a notable proportion of medical inaccuracies that occur in neurosurgery are technical in nature, and pertain to the surgical procedures themselves, which can be obviated. This underscores the significance of practical measures intended to enhance the positive result of neurosurgical interventions, and diminish related inaccuracies, with the ultimate goal of delivering optimal healthcare to patients. Recent technological advancements have narrowed the divide between machines and humans, and have empowered computers to emulate, and surpass, innate human intelligence, thus resulting in the emergence of AI as well robotics, VR, and AR (7, 8).

In this study, we aimed to review the role of AI, VR, robotics, and AR in neurosurgery and clarify the promising perspective of neurosurgery with the help of the aforementioned technologies.

### Artificial intelligence and neurosurgery

The utilization of computer systems to stimulate critical thinking and intelligent behavior was originally expounded upon by Turing in 1950 (9). Six years later, McCarthy provided an explanation of artificial intelligence, outlining it as the engineering and science of generating intelligent machines (10, 11). As time progressed, the development of AI through the use of more intricate algorithms resulted in performance that more closely resembled that of the human brain. Within the field of medicine, two subfields of AI, namely DL and ML, have emerged with significant roles. In ML, pattern identification is utilized to enable the analysis of specific situations, allowing for subsequent learning and the application of acquired data to future same scenarios. Also, this tool can be leveraged in the context of individualized patient care and clinical decision-making. DL, on the other hand, represents an advanced form of ML that operates more closely to the human brain. Algorithms are employed to establish an artificial neural network (ANN) that is able to make decisions and learn autonomously (12–14). In the past five decades, both DL and ML have played a noticeable part in the advancement of AI in the field of medicine. The utilization of predictive models has facilitated medical diagnosis, prediction of therapeutic responses, and preventative medicine (15). The employment of AI has resulted in a reduction of errors and costs of care, and has provided valuable context for patient care, thus resulting in several benefits (16).

AI is capable of enhancing the precision of treatment and diagnosis in the field of neurosurgery, while also providing neurosurgeons with timely and effective tools for pre-, post-, and intraoperative care. Neurosurgeons benefit from AI's ability to detect subtle malformations and abnormalities from clinical data as well as neuroradiological images that may elude even highly-trained human eyes. DL, a type of ML, utilizes neural networks with multiple layers of learning algorithms (17).

In the pre-operative phase of neurosurgical procedures, artificial intelligence (AI) can provide valuable assistance to surgeons by aiding in the diagnostic process, selecting appropriate patients for treatment, and guiding patients towards informed decisions (18). During the intra-operative phase, the technology of AI significantly improve the surgical performance of neurosurgeons as well as help to minimize the occurrence of errors in their procedures. In the postoperative phase, AI is utilized to accurately predict patient's prognosis, identify potential complications that may arise after surgery, and track pertinent data that is used to enhance the quality of aftercare and patient recovery. By leveraging the predictive capabilities of AI in the postoperative phase, pre-operative planning can be optimized to facilitate better patient care and decrease overall related costs. For instance, machine learning techniques can be employed to classify, regress, and cluster large data sets, thereby enabling the identification of risk factors and the prediction of surgical complications including cardiac and wound-related issues, as well as mortality rates among patients undergoing cervical discectomy as well as posterior lumbar spine fusion procedures (19, 20).

The utilization of ubiquitous and high-resolution radiological imaging in combination with electrophysiological data has become the preferred methods for providing neurosurgeons with unparalleled and noninvasive access to intracranial regions. In the field of neurosurgical medicine, effective decision-making requires the careful study, retention, analysis, and interpretation of a large quantity of complex and dynamic data. Typically, neurosurgeons rely on their clinical expertise and empirical evidence to formulate decisions and predict prognoses (21, 22).

The potential of AI in predicting the disease progression has been demonstrated through the use of DL algorithms trained on magnetic resonance imaging (MRI) data from a large, multi-institutional dataset. This approach has shown promise in replacing the need for invasive tissue sampling in predicting the progression of glioma in a non-invasive manner. The application of ML in this context has the potential to enhance the capitalization of existing data (23, 24).

In the context of temporal lobe epilepsy (TLE), as the most prevalent surgically remediable and pharmacoresistant type of epilepsy among adults, the performance of artificial intelligence (AI) has been found to surpass that of physicians. Specifically, AI demonstrated a 95.8% success rate in lateralising the influenced brain hemisphere, as opposed to the 66.7% demonstrated by physicians, when utilising functional MRI data (25). This outcome is of particular significance, as an uncertain localisation of the epileptogenic zone is able to pose a noteworthy challenge in terms of allocating patients who are eligble to proper surgeries. Therefore, the utilization of AI in this context may have the potential to greatly enhance patient outcomes (26).

There exist additional instances in which artificial intelligence (AI) was utilized for the categorization and diagnosis of neurosurgical issues without the aid of radiological input. Specifically, AI exhibited a substantially heightened accuracy in discerning between single cells vs. multiunit spike clusters from electroencephalography recordings of twelve epilepsy patients who necessitated the implantation of chronic intracranial depth electrodes (27, 28). Due to its ability to concurrently utilize multiple variables, a capability that surpasses that of a human operator, AI can take into account numerous factors when planning treatment. As such, a study was conducted, which involved the creation of an artificial neural network, comprising of eleven clinical inputs, in order to train the algorithm for the survival rate prediction of patients with traumatic brain injuries (TBI). In addition, the performance of ML in terms of accuracy and sensitivity was superior to that of neurosurgeons and neurosurgery residents, and it was also more specific (29).

However, it is nothworthy that AI, ML, and DL are not imbued with any mystical properties. Rather, they represent a set of advanced statistical algorithms and mathematical models (which frequently depend on recursive functions) that can now be readily incorporated into everyday applications owing to the augmentation of computational capabilities. In continue, we will disscuss some examples of how AI can help in pre-, post-, and intraoperative care:

• Pre-operative phase: during the pre-operative phase of neurosurgery, AI has the potential to provide aid to surgeons in diagnosing the condition, the determination of patients for the appropriate treatment, and the facilitation of informed decision-making by patients (18). AI algorithms have been employed for automated neoplasm segmentation, localization of epileptogenic zones, identification of suitable candidates for epileptic surgery, prognostication of symptomatic cerebral vasospasm following aneurysmal subarachnoid hemorrhage, as well as estimation of tissue damage post-acute ischemic stroke (8). For instance, the categorization of tumor and epilepsy can be subjective, thereby leading to disparities in the decision-making among neurosurgeons. Upon preparing a robust outline and framework, algorithms utilizing AI can mitigate the subjective interpretation of the data and consequently diagnose medical conditions necessitating neurosurgical procedures (30, 31).
• Intra-operative phase: during the intra-operative phase of neurosurgical procedures, AI has the potential to amplify the surgeons' performance and mitigate some errors that are commonly encountered during neurosurgical procedures (31).
  The current traditional approach to performing intraoperative tissue biopsy involves transporting the tissue to a laboratory, processing it, and preparing specimens with the assistance of skilled laboratory professionals before pathologists interpret the results. This process has been in use for over a century and is both time-consuming and resource-intensive. However, there have been recent developments in the utilization of AI technology during the intraoperative phase of neurosurgery. For instance, Hollon et al. designed a label-free optical imaging workflow that can predict diagnosis of tumours in approxiamately real-time automatically. The tumor diagnosis techniques are able to predict the tumour diagnosis in less than 150 s, which is significantly faster than conventional methods that can take up to 30 min. Furthermore, their overall accuracy rate of 95% is marginally better than regular histology workflow, which has an accuracy rate of 94% (32–34).
• Post-operative phase: given that patients may necessitate multiple visits to different geographic locations like inpatient wards, outpatient clinics, pharmacies, intensive care units, emergency departments as well as laboratories, telemedicine possesses the capacity to curtail unnecessary travel for both patients and healthcare professionals (35, 36). The implementation of telemedicine services is held in high regard by both healthcare providers and patients and has the potential to enhance patient outcomes in the postoperative phase, particularly in regions with limited geographic access. The majority of patients welcomed postoperative videoconferencing which was found to be as effective and safe as in-person clinic visits for those who had elective neurosurgery (37).

### Robotics and neurosurgery

Robotics, a fast-moving discipline, is transforming neurosurgery practice with advances in machine learning and artificial intelligence. Utilizing robotics in neurosurgery can efficiently omit mechanistic errors, decrease operation time, and prepare more extended respective margins using minimal-access operation. In this way, minimal complications and great surgical results will be achieved (3). Interestingly, it was reported that the first use of robotics in operation was a neurosurgical biopsy. The Unimation PUMA (Programmable Universal Machine for Assembly) 200 robot was utilized in a 52-year-old man to position a needle guided by a CT scan in a stereotactic biopsy of an intracerebral lesion (38). Then, the aforementioned robot was used as an assistant to retract delicate neural structures while resecting low-grade thalamic tumors among children (39). NeuroMate robot was the first FDA-approved device specifically generated for neurosurgical use (3).

Current available robotic systems used in surgery have three subtypes: master-slave, semi-active, and active (40). Active robotic systems can work autonomously and perform preprogrammed tasks. However, master-salve systems depend on surgeon input and lack preprogramming. Semi-active ones are hybrid in which surgeon inputs complement preprogrammed elements of the system (41). Improved visualization for surgeons, greater precision, as well as a decrease in fatigue are some benefits of using robotic systems in surgery (42). Regarding limitations, there are some concerns about cost, hardware maintenance, and sterilization (43).

Generally, robotic systems can be used in neurosurgery for procedures with restricted operative spaces. Anatomical localization, surgeon's hand stabilization, placement of pedicle screws in spinal procedures, and plans to access deep brain targets are some robot applications in neurosurgery (43–45). Pathfinder, SpineAssist, Renaissance, Neuromate, and NeuroArm are common robotic systems utilized in neurosurgery (43, 46). Robotic assistant is more common among other surgeons, but specific aspects of neurosurgery including the technical and microsurgical nature of procedures as well as the history of its innovation in stereotaxy help it for being well incorporated with robotic assistance (45).

In 2022, Singh et al. claimed that the usage of robotic systems in neurosurgery is in its infancy yet. Almost 30 per 100 neurosurgical departments use robotic cranial methods and 40 per 100 departments use robotic spinal methods. While examining the possible application of robotic systems in neurosurgery, 13 clinical trials seemed to be applicable, and none of them were completed (47). Various indications for robotic usage during neurosurgery are identified. For instance, multiple studies claimed that screw placement assisting by robots during spinal surgery is accurate and safe (48–50) and cause less radiation exposure as well as fewer facet joint violations while screw placement in comparison to traditional surgery methods (51, 52). Three different systematic reviews and meta-analyses reported that the usage of robotic systems can lead to a high accuracy compared with conventional free-hand strategies (53–55).

In continue, we elucidate some examples of robotic systems used in neurosurgery in detail:

• The telesurgical robot: In this particular variety of robot, surgeons exercise remote control over the robot's actions. The NeuroArm, hailing from the University of Calgary in Canada, displays remarkable potential. It constitutes an MRI-compatible robotic arm that emulates a surgeon's manual gestures. It harnesses piezoelectric motors and boasts of eight degrees of freedom (DOF). This technology has undergone continuous development, with bespoke microsurgical instruments (equipped with force-sensing as well as force-calibration features) recently incorporated into the robotic arm's arsenal. Encouraging preliminary experiments conducted on rats have paved the way for its subsequent deployment on human subjects. Additionaly, It is the first robot to furnish the neurosurgeon with tactile feedback while simultaneously being operated remotely from a workstation located outside the operating room. Reports indicate that it has already been employed in over 1,000 neurosurgical methods, such as MRI-guided tumor biopsies, hematoma evacuations, and microsurgical dissection (43, 56, 57).
• The supervisory surgeon-controlled robot: the robotic system supports surgeons in executing accurate procedures. The PUMA robots, introduced in the 1980s, have become the most prevalent neurosurgical robots to date. Additional robots, such as the Pathfinder and Minerva robots, have been subsequently developed. These robots mainly undertake stereotactic duties, without or with a frame, and have advanced from directing biopsy needles as well as depth electrodes to inserting and devising pedicle screws in the spine (3).
• Handheld shared/controlled systems: The collaboration between surgeons and robots occurs at the site where they jointly dissect and manipulate the structures of brain through instruments. This allows the precise robot actions to complement the manual dexterity and manipulative skills of neurosurgeons, resulting in a synergy of capabilities. It can be likened to the optimal combination of two distinct worlds. The Steady Hand System, developed at John Hopkins University, is a representative instance of the few systems currently in development. This instrument, which is held by both the surgeon and the robot, permits finer dissection and eliminates tremor and muscle fatigue. Other devices, such as the Evolution 1, can be controlled for endoscopic procedures. The NeuRobot, developed at Shinshu University in Matsumoto, Japan, is a remotely operated device that comprises an endoscope equipped with twin tissue forceps, which can assist in tumour resection (3).

## Augmented and virtual reality in neurosurgery

### Virtual reality

Virtual reality (VR) is a process that entails the user's immersion in a system obscuring the natural world as well as generating a virtual realm for users' experience. VR can be classified as either immersive or non-immersive, based on whether the virtual world is generating as a powerful substitute for the real world or virtual environment, respectively (58).

**Figure 1:**
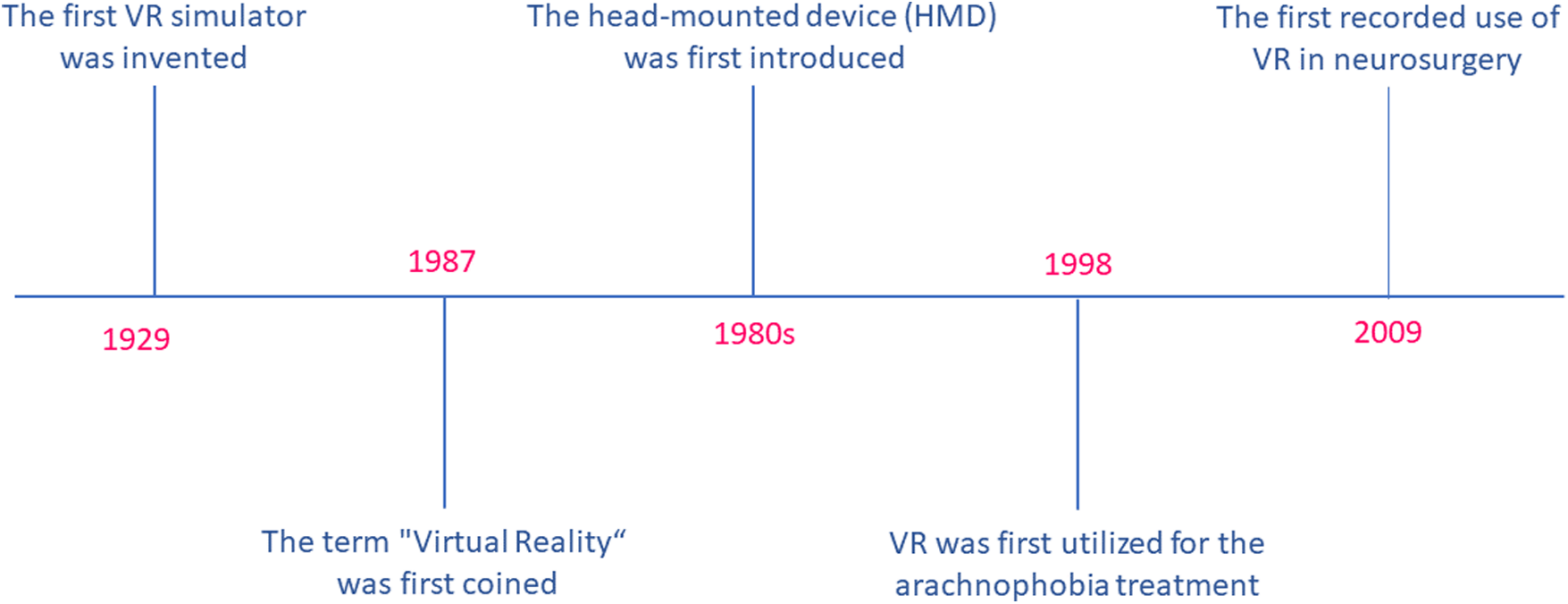
Virtual reality-related timeline.

Although the concept of VR was utilized for panoramic viewing as early as the eighteenth century, it was not until 1929 that the first VR simulator, specifically a flight simulator, was invented. However, the term “Virtual Reality” was not coined until 1987 (59). The development of VR technology can be traced back to the innovative contributions of Tom Furness, an electrical engineer, who was affiliated with the United States Air Force (60). Furness's contributions were groundbreaking and earned him the moniker of “The Godfather of Virtual Reality” (61). The introduction of a VR system in the field of medicine was pioneered by Robert Mann in the field of orthopedics for the first time. Subsequently, the head-mounted device (HMD) was introduced in the 1980s. Although VR had been utilized for the arachnophobia treatment in 1998, this marked the first reported use of the technology in pathology treatment (62, 63). It is noteworthy, however, that the first recorded use of VR in the therapy of neurosurgical disorders is a recent occurrence. For the first time, Clarke utilized the NeuroTouch neurosurgical simulator to excise a Left frontal meningioma in 2009 (see Figure 1) (64).

Throughout the literature, the terminology of Virtual Reality has been utilized interchangeably to encompass AR and Mixed Reality (MR). Anyway, it is crucial to note that MR, AR, and VR are fundamentally distinct technologies. VR specifically pertains to computer-generated three-dimensional (3D) immersive environments, while AR involves the projection of computer-developed images onto real-world images. On the other hand, MR entails the projection of virtual objects into the physical world, where the objects demonstrate spatial awareness and responsiveness (65). The early usage of VR technology yielded adverse effects such as temporarily impaired vision, a lack of sense of presence, vomiting, and nausea. However, these limitations were primarily attributed to the technical constraints of the VR technology available during that time, similar to the human eye's inability to focus in-depth on a 3D-rendered image (65–67). Similarly, high-definition 4 K 3D exoscopes have also been reported to produce similar limitations during surgical procedures (68, 69).

Cerebrovascular and neuro-interventional surgeries heavily rely on advanced neuroimaging strategies for operative prognostication and decision-making. The clinical use of VR enhances the diagnostic efficacy and accuracy of the aforementioned techniques (70). Hybrid angio-suites enable neurointerventionists to create an immersive VR model according to patient-specific anatomy, which improves crisis resource management, training, and procedural skills (71). VR technology has been of great utility due to its specific metric-based performance assessment which is outside the angio suite as well as its ability to conduct complex neuro interventions, such as mechanical thrombectomy, accompanied by the similar set of principles as in live patients (72). Surgeons can also benefit from gain access to a VR-based patient-specific model for better planning management or diagnosing strategies, along with planning complex hybrid or combined procedures requiring a combination of conventional and interventional surgical methods (73–75).

A primary obstacle encountered by individuals training to become neurosurgeons is performing procedures with bimanual dexterity within a narrow corridor that is bound by intricate and essential non-resilient bones and neurovascular structures. Neuronavigation is highly relied upon by those in training for better planning their localization and approach. However, it is not an appropriate means of advancing spatial reasoning abilities, also an excessive dependence on neuronavigation can hinder the skills development (58). Additionally, as the operation progresses, the brain shifts gradually, rendering the preoperative imaging used in the navigation system less accurate and useful. To circumvent this issue, an intraoperative brain imaging system (IBIS) was created that recognized any discrepancies between preoperative imaging and intraoperative ultrasound. Through the use of IBIS, intraoperative stimulation is altered in real-time, as well as inaccuracies are updated using AR (76, 77). The utilization of VR in training and simulation has proven to be a superior alternative in reducing operative stress duration and cognitive load, as well as enhancing efficacy for novice neurosurgeons, according to a study (78). There is a diverse range of available VR tools for neurosurgical training and education, including a multifunction head-mounted display (HMD) such as Microsoft HoloLens and Google Glass, in addition to haptic feedback tools such as Procedicus Vascular Interventional System Trainer (VIST), Immersive touch, NeuroVR, and synthetic tissue simulators like SynDaver, Creaplast, Thomas Jefferson University Durotomy Repair Module, and iDU optics 3D-printed models. Additionally, there are VOSTARS (video and optical see-through AR surgical systems) HMD-based surgical navigation platforms, as well as operating planning devices such as Dextroscope, Surgical Theatre, Synaptive Medical, and VPI Reveal. The use of computer simulation and VR has extended to various fields, including pilot training, medicine, and military, as a means to alleviate potential dangers by preparing a virtual simulator as well as haptic and visual feedback (79–81). Physics-based simulators pose a challenge due to their high computational demands and requisition of resources, both in terms of computing skills and software, to prepare haptic and visual feedback, along with formal trainings in 3D immersive simulation (79, 82, 83). Amongst the various displays in virtual reality (VR), HMDs offer the greatest engagement, with other displays such as Google Glass featuring an OLED or LED display with a high refresh rate of 120 Hz as well as latency time of approximately 20 milliseconds (84). Additionally, VR plays a crucial role in tele-proctoring, facilitating the training of surgeons on complex techniques and procedures, independent on their geographical location (85). Also, immersive technologies have a profound effect on global virtual connections, enabling middle- and low-income countries to enhance their potential applications particularly while ongoing pandemics, like COVID-19. In future outpatient neurosurgery consultations, telemedicine is expected to have a crucial role as it facilitates the interaction between the surgeon and patients in a “merged reality” space, thereby enabling manual, visual, and verba interactions between them. While utilizing VR technology as an educational tool for neurosurgeons, specific quality control standards must be followed, including appropriate sound quality, high-resolution images, internet speed, high processing power, visual and haptic feedback, tissue fidelity, and organ structure. The main benefits of using VR technology in the field of neurosurgery training over animal and human models are its non-invasive nature, low cost, limitless repetition ability, as well as the extensive diversity and variety in cases which can be simulated. By the way, the ever-present concern is the realism and resolution of the VR technology (86–89). The employment of VR environments presents an opportunity to accurately gauge the performance of surgeons, evaluate their proficiency, and monitor their progression during training. In addition, the implementation of AR HMD visualization has been shown to elicit greater levels of enthusiasm and enjoyment in the learning process, particularly among younger surgeons (90–92).

### Augumented reality

AR is a novel technological advancement that overlays 3-D virtual text or objects onto tangible objects (93). Divergent from VR, which generates a wholly fabricated environment, AR presents both virtual and tangible objects, thereby producing a semi-immersive experience for users. Giglioli et al. claimed that AR amplifies user perception of reality by integrating virtual content into the tangible world and displaying it simultaneously and in real-time. Additionally, they elucidate that AR encompasses an array of tools and methods that supplement physical reality with additional information (94). The implementation of AR technology in healthcare has been adopted by the field of neurosurgery at an early stage. This particular medical specialty depends highly on imagery for the purposes of preoperative planning and intraoperative neuronavigation. The present neuronavigation system lonely projects 2-D images (coronal, sagittal, and axial) on a computer screen, as explained by Pandya et al. In order to successfully navigate the 2D images into a 3D format, the surgeon must engage in a mental transformation and be able to project the visualized data onto the patient's view. However, this task creates a significant interruption in the surgical workflow as the neurosurgeon must frequently switch between the computer screen and the surgical field (70, 95).

The American Brain Tumor Association has reported that in 2013, the Central Brain Tumor Registry of the United States approximated 69,720 novel cases of primary brain tumors. Johns Hopkins Medicine suggests that the primary objective of operation for metastatic brain tumors is to remove and debulk the whole tumor during simultaneously preserving neurological function (96). Currently, the use of image-guided neurosurgery (IGNS) plays a critical role in achieving maximal brain tumor resection. Deng et al. have elucidated that IGNS utilizes patients'preoperative images to track the tumor's position against the preoperative images while surgery. Nevertheless, they have postulated that by using IGNS, surgeons must switch views between the surgical field and the computer screen every time he/she desires to control his/her relative position on the preoperative images and patient's brain (97). The division of the preoperative images on the computer screen into three distinct images (coronal, axial, and sagittal) necessitates the surgeon's mental amalgamation of these images to create a singular three-dimensional composite image. It has been posited that the repeated alteration of perspectives while surgery hinders the surgeon's workflow. Currently, numerous AR system prototypes are undergoing testing, specially for brain tumors management. Inoue et al. employed an AR system prototype together with a web camera in order to superimpose the brain tumor images in the patient's dura and skull. Similarly, Deng et al. utilized a wireless tablet computer AR neuronavigation system for operative planning and the execution of two cases in China (95, 97, 98).

Abe and colleagues conducted an experimental study on a virtual protractor with an AR system, known as VIPAR, for percutaneous vertebroplasty. The study involved the use of 5 patients and 40 spine phantoms. VIPAR was developed to provide real-time visualization of the vertebroplasty needle trajectory in a 3-D space while the procedure. Generally, percutaneous vertebroplasty is a minimally invasive procedure that is aimed at treating fractured spinal vertebrae that cause loss of function and pain. This procedure involves injecting medical grade cement into the fractured vertebra, and it significantly depends on the utilization of C-arm fluoroscopy in order to guide neurosurgeons in controlling the needle trajectory. Also, Abe et al. emphasized that while percutaneous vertebroplasty is generally considered a safe and almost easy procedure, incorrect needle placement can lead to cement leakage and neurovascular injury. Johns Hopkins Medicine has proffered that there are various risk factors which are related to vertebroplasty, such as rib or other surrounding bone fractures, hemorrhaging,, as well as cement leakage outside the bone. Upon conducting 40 spine phantom trials, Abe et al. discovered that the error of the insertion angle of the vertebroplasty needle while procedure was highly improved in comparison to present modalities. Furthermore, in these 5 VIPAR assisted percutaneous vertebroplasty procedures conducted in the clinical trial, there was a complete success rate, with no spinal pedicle breach or leakage of cement (99, 100).

## Limitations

In the field of health care and medicine, AI has made significant progress. In the future, doctors and robots may collaborate to improve patient care. Nevertheless, patients may find it challenging to place their trust in a robot when it comes to surgical procedures, and it is often recommended that a neurosurgeon retain ultimate control. Traditional neurosurgeons typically dissuade the use of aforementioned technologies including AI during neurosurgical interventions. Conversely, an excessive reliance on AI may deter surgeons, particularly neurosurgeons, from mastering the necessary surgical skills (31, 101). For instance, AI necessitates an extensive dataset for its operation, thereby presenting the challenge of generating a plethora of clinically practical algorithms. This entails the storage of large-scale data, allowing for easy accessibility to abusers, thereby jeopardizing patient privacy. Numerous ethical concerns arise in this realm. Although the recording of patient data remains controversial, in the event of a misdiagnosis due to AI, moral and legal quandaries require prompt attention (102). The “black box dilemma” emerges, where both consumers and users lack comprehension concerning how the computer produces outcomes, ultimately hindering transparency in AI systems (103). One must also acknowledge that, regardless of how advanced AI becomes, it lacks human consciousness and the capacity to make conscientious and informed decisions (104).

It is highly recommended to certify and verify AI-based systems with a view to ensuring the safety of patients. Moreover, it is imperative to minimize the instances of AI system failures on patients. An additional challenge that looms ahead is the annotation of targets, given that the identification of anatomical structures can be a daunting task for even the neurosurgeons. In order to address this challenge, AI needs to be trained to recognize such intricate anatomy, in conjunction with other cutting-edge technologies, thereby enhancing accuracy in dealing with difficult targets. However, the fact remains that the bulk of data in a training set is dominated by standard cases, which makes cases with anatomical abnormalities a worrisome challenge for the future. In the context of endovascular procedures, AI is constrained by the lack of haptic feedback, which limits its potential for usage (102, 105). Nonetheless, AI can be extensively leveraged in surgeries for the elderly, but it is still incumbent upon the clinician to provide the necessary endorsement. Doctors must therefore acquire a working knowledge of computer science in order to effectively analyze and optimize the data and AI systems at their disposal (106, 107).

## Conclusion

The field of AI in corporations with VR, AR, and robotics is an interdisciplinary area located at the interface of medicine, neuroscience, and engineering. In the realm of neurosurgery, they possess the potential to optimize patient outcomes. In the pre-, intra-, and postoperative stages of neurosurgery, they have the ability to enhance surgeons’ skill sets. The recent technological advances in AI, VR, AR, and robotics have made it possible for humans and machines to collaborate to improve healthcare delivery. This is achieved via image acquisition, processing and interpretation, patient allocation to appropriate surgeries, intra-operative improvements, postoperative follow-up, as well as facilitating access to high-quality healthcare. However, more investigations are required to better evaluate the limitations. Also, the possibility and accessibility of the wide use of these techniques must be evaluated.
